# Supplementary material for: The PHD finger of Arabidopsis SIZ1 recognizes trimethylated histone H3K4 mediating SIZ1 function and abiotic stress response
Source: Commun Biol. 2020 Jan 10;3:23. doi: 10.1038/s42003-019-0746-2 (PMC6954211; doi:10.1038/s42003-019-0746-2)
Supplement: Supplementary file 1 — Supplementary Information [file 42003_2019_746_MOESM1_ESM.pdf]

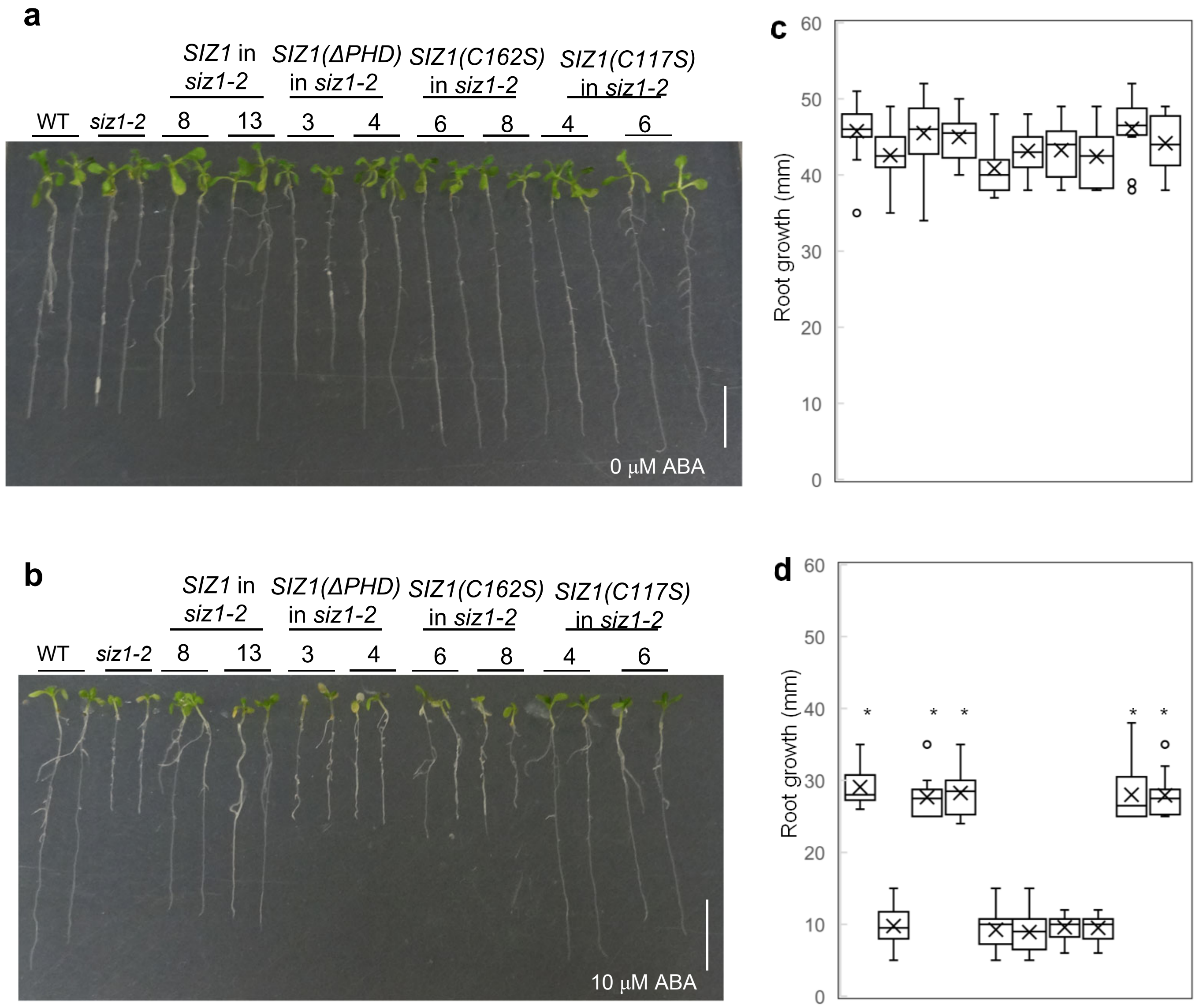

Supplementary Figure 1. *SIZ1* with mutation in the PHD finger was unable to complement ABA inhibition of primary root growth in seedlings of the *siz1* mutant. Three-day-old seedling were transferred onto the basal media containing 0 (**a**) or 10 µM ABA (**b**). Photographs are of representative seedlings 6 days after transfer onto the media. The bar indicates 1-cm length. Root growth on the media without (**c**) or with 10 µM ABA (**d**). was measured. The order of the values are same as Supplementary Fig. S1a or S1b. The values are mean ± standard deviation (SD; n = 12). Asterisk indicates a significant difference from the *siz1-2* plants ($p < 0.05$) as determined by Student's *t*-test.

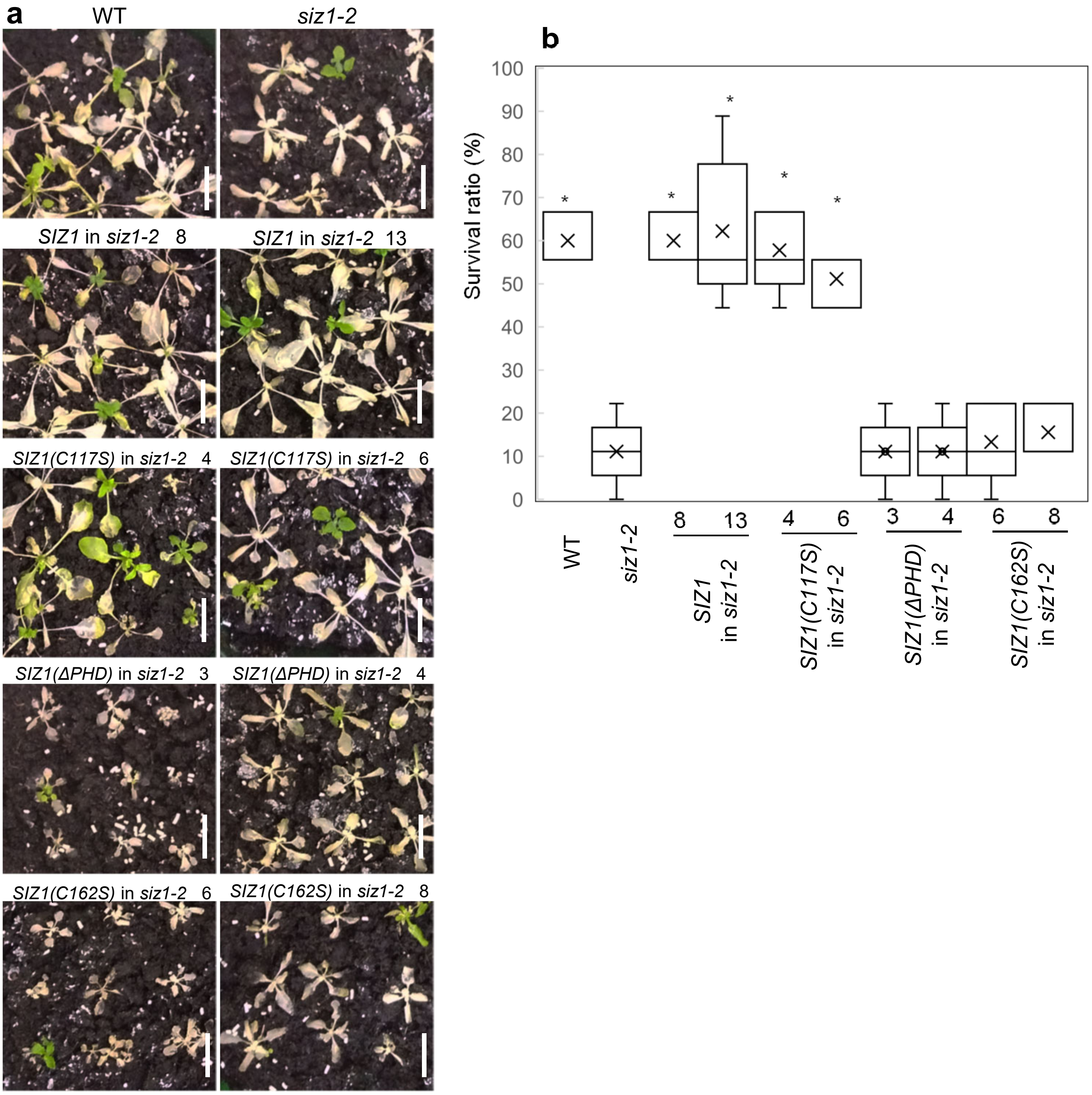

Supplementary Figure 2. *SIZ1* with mutation in the PHD finger was unable to complement cold sensitivity of the *siz1-2* mutant. **a**. Three-week-old plants were incubated at 4ºC for 1 week for cold acclimation. Cold-acclimated plants were exposed for 4 h at -7ºC. After freezing treatment, plants were incubated at 4ºC for 1 day and done at 24ºC for 1 week. Photographs are representative of WT, *siz1-2*, and *siz1-2* transformed with *SIZ1* variants. **b.** Survival was determined. Data are the mean ± SD calculated from four independent experiments. Asterisks indicate a statistical difference from the *siz1-2* plants ($p < 0.05$) as determined by Student's *t*-test. The bar indicates 2-cm length.

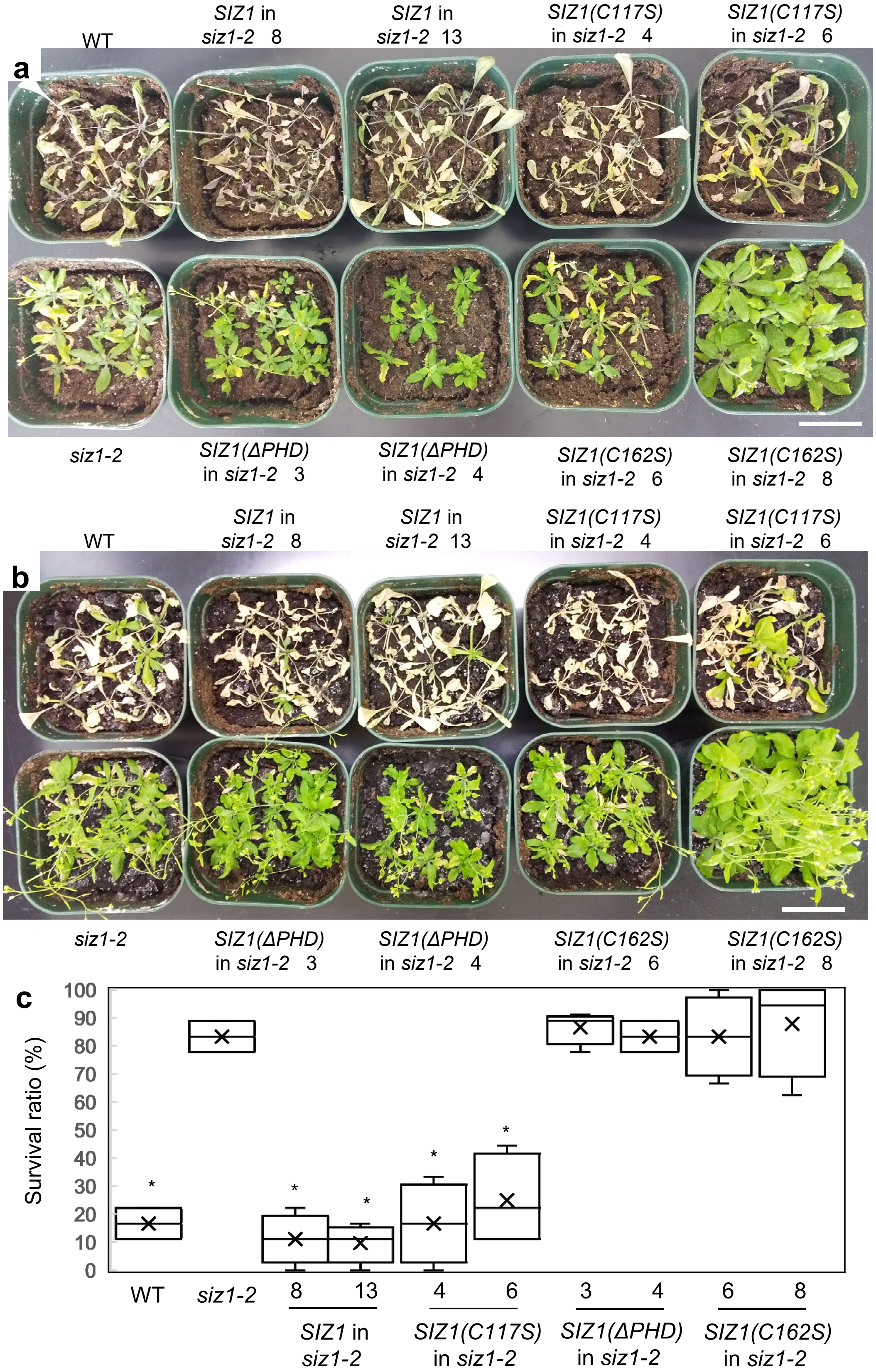

Supplementary Figure 3. *SIZ1* with mutation in the PHD finger was unable to complement drought tolerant phenotype of the *siz1-2* mutant. **a,** Water was withheld from 2-week-old plants for 18 days. **b,** Watering was resumed, and plants were incubated for 1 week. **c,** The survival ratio was determined for 9 plants after drought treatment. Data are mean ± SE from four independent experiments. Asterisks indicate a statistical difference from the *siz1-2* plants ($P < 0.05$) as determined by Student's *t*-test. The bar indicates 3-cm length.

Supplementary Figure 4 (unprocessed blots)

Figure 6a

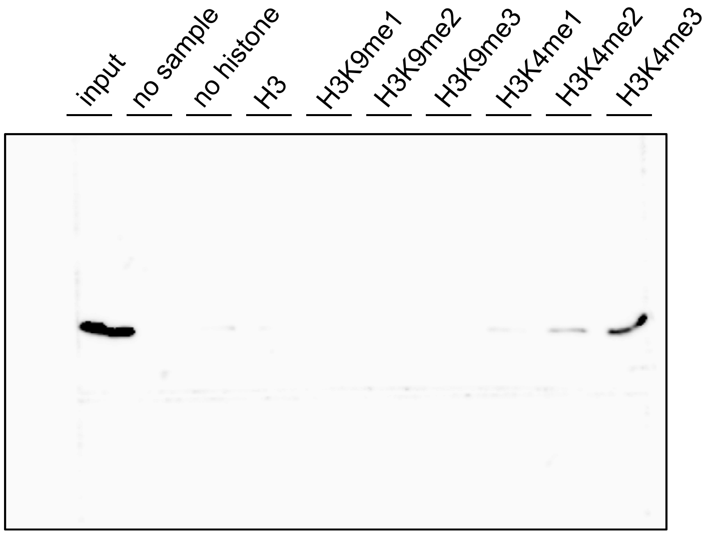

Figure 6b

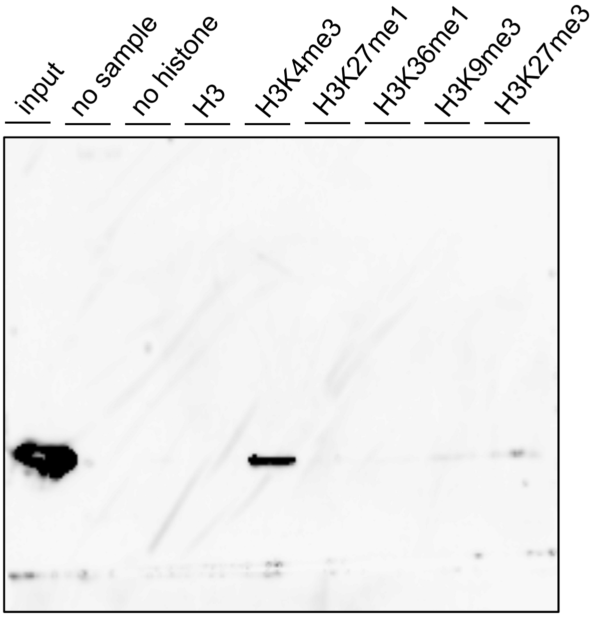

Figure 6c

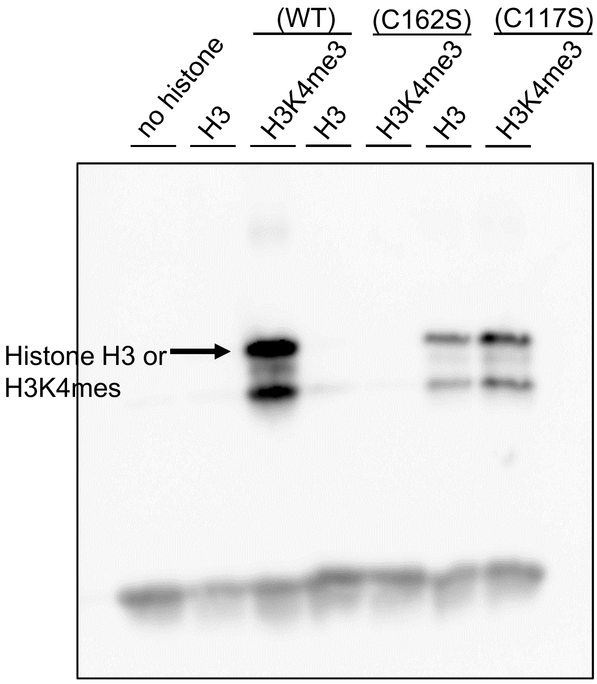

Figure 6d

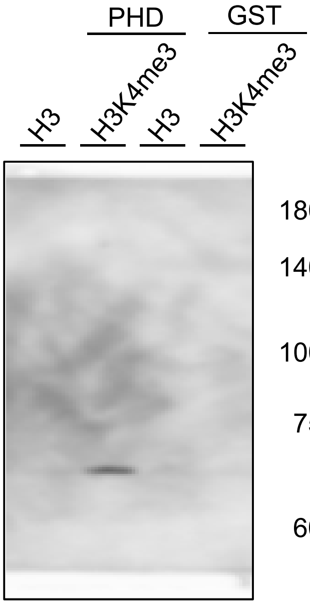

Figure 8b

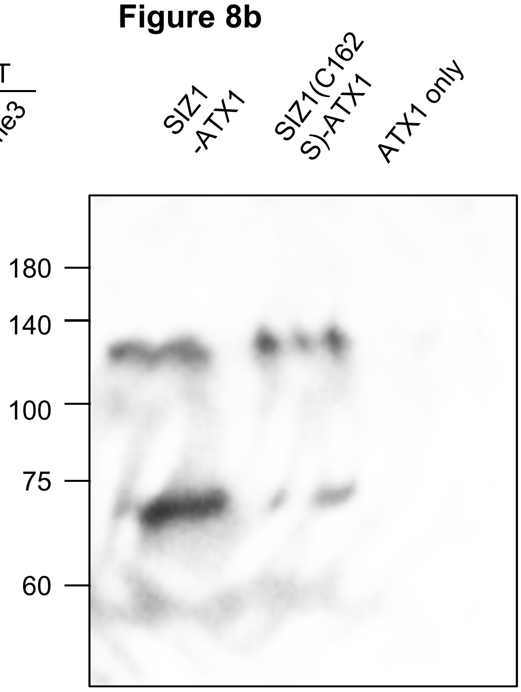

Supplementary Table 1. Primers used for plasmid construction

Name of primer	DNA sequence (5'-to-3')
**pCambia1302-AtSIZ1full::AtSIZ1(ΔPHD):GFP**	
SIZ1-2hyF	GCTAGCATGGATTTTGGAAGCTAATTG
SIZ1-2hyR	CACTAGTTTACTCAGAATCCGAGTCAAT
SIZ1-PHDdeltaF	CAACCAGAAATTAAAGTTCGACGACTTA
SIZ1-PHDdeltaR	TGGGTCAGCTCGAGTAAGTCGTCGAACT
**Detection of *siz1-2* mutation**	
LP034008	CTGATGGTAGCCTTTGCCCCT
RP034008	AAACCCGACTGAGCTGAAGCA
Salk_LBa1	TGGTTCACGTAGTGGGCCATCG
**pCambia1302-AtSIZ1full::AtSIZ1(C162S):GFP**	
AtSIZ1-C162S-F	CATTTTATTCTGAAATCTGCCG
AtSIZ1-C162S-R	CGGCAGATTTCAGAATAAAATG
**pCambia1302-AtSIZ1full::AtSIZ1(C117S):GFP**	
AtSIZ1-C117S-F	CGATGTGTTTCAGGAAACTCGC
AtSIZ1-C117S-R	GCGAGTTTCCTGAAACACATCG
**GST-PHD**	
pGEX5X-PHD-F	TGGGATCCCCGAATTCTGTGTTTGTGGAAACTCGCT AGAAAC
pGEX5X-PHD-R	GGCCGCTCGAGTCGACGCAGATTTCACAATAAAAT GATTCC
**pMAL-ATX1SET**	
ATX1SET-F-SalI	TAGAGTCGACAGGGAAACATACAGGAAGAG
ATX1-SalI-R	CCGAGTCGACTTATTCTGCGGTCCAGTC
**pMAL-ATX2SET**	
ATX2SET-F-SalI	AACCGTCGACAAGAGATTAGCATTCGGGAA
ATX2-SalI-R	CCGAGTCGACTCAGGACTCTGTCCACTC
**pMAL-ATX3SET**	
ATX3SET-F-BamHI	ATTCGGATCCCGGCTAAAACATTTGCAGAG
ATX3-BamHI-R	CCGAGGATCCCTAGTTCATGAATTTGCG
**pMAL-ATX4SET**	
ATX4SET-F-BamHI	TTTCTGGATCCAGGGAACGACTTCACC
ATX4-BamHI-R	CGAGGATCCCTAATTCATGAATTTCCTGCA
**pMAL-ATX5SET**	
ATX5SET-F-BamHI	TTTCCGGATCCAGGGAACGGCTTCACC
ATX5-BamHI-R	GAGGATCCATTCATGAATTTCCTGCAGTTG

Supplementary Table 1. (continued)

Name of primer	DNA sequence (5'-to-3')
**pBYR2HS-CFH**	
pBYR2HS-Flag-F	ACTGTTGATAGTCGACATGGACTACAAGGACGATGACGACAAGG
FLAG-His	CAAGGACGATGACGACAAGGTCGAGATGGATTATAAGGATGACGATGACAAGGTTGAGCATCACCATCACCA
pBYR2HS-FlagHis-R	ATTCAGAATTGTCGATTAGTGGTGGTGATGGTGATGCTCAAC
**pBYR2HS-CRH**	
pBYR2HS-HRV3C-F	ACTGTTGATAGTCGACCTTGAGGTTTTGTTCCAGGGTCC
HRV3C-RAP-His	CTTGAGGTTTTGTTCCAGGGTCCTGACATGGTGAATCCTGGTCTTGAGGATAGGATCGAGCATCATCACCACCATCACTAA
pBYR2HS-stopHis-R	ATTCAGAATTGTCGATTAGTGATGGTGGTGATGATG
**pBYR2HS-AtSIZ1-FH**	
pBYR2HS-AtSIZ1-F	CACTGTTGATAGTCGATGGATTTGGAAGCTAATTGTAAGG
pBYR2HS-AtSIZ1-R	TGTAGTCCATGTCGACCTCAGAATCCGAGTCAATGGAGAGG
**pBYR2HS-ATX1-RH**	
pBYR2HS-ATX1-F	CACTGTTGATAGTCGATGGCGTGTTTTTCTAACGAAACCC
pBYR2HS-ATX1-R	AAACCTCAAGGTCGACTTCTGCGGTCCAGTCTATTAGATCAC

Supplementary Table 2. Primers used for detection of transcripts and for ChIP analysis

Name of primer	DNA sequence (5'-to-3')
SIZ1	
LP023805	GCTGACGTTTCAGGAGGTTTAGTTG
RP023805	GCCTTGTCTTGTCTACTGTCATTCATAC
**WRKY70-ChIP**	
WRKY70-ChipF	CTTCAAACTTGCCGTCGTTA
WRKY70-ChipR	ATCTCCTCCTCCTCATCCCT
**pCambia1302-AtSIZ1full::AtSIZ1(C162S):GFP**	
AtSIZ1-C162S-F	CATTTTATTCTGAAATCTGCCG
AtSIZ1-C162S-R	CGGCAGATTTCAGAATAAAATG
**pCambia1302-AtSIZ1full::AtSIZ1(C117S):GFP**	
AtSIZ1-C117S-F	CGATGTGTTTCAGGAAACTCGC
AtSIZ1-C117S-R	GCGAGTTTCCTGAAACACATCG
